# HTLV-1 in Pregnancy and Neonatal Health: Evidence, Challenges, and Future Directions

**DOI:** 10.3390/diagnostics15151886

**Published:** 2025-07-28

**Authors:** Ana Clara Assis Alves Emerick, Letícia Castilho Yamanaka, Stefany Silva Pereira, Tammy Caram Sabatine, Taline de Brito Cavalcante, Thamy Cristina Campos, Gustavo Yano Callado, Edward Araujo Júnior, Antonio Braga, Gloria Calagna, Evelyn Traina

**Affiliations:** 1 Discipline of Woman Health, Municipal University of São Caetano do Sul (USCS), São Caetano do Sul 09521-160, SP, Brazil; ana.alves1@uscsonline.com.br (A.C.A.A.E.); leticia.yamanaka@uscsonline.com.br (L.C.Y.); stefany.pereira@uscsonline.com.br (S.S.P.); tammy.caram@uscsonline.com.br (T.C.S.); taline.cavalcante@uscsonline.com.br (T.d.B.C.); thamy.campos@uscsonline.com.br (T.C.C.); araujojred@terra.com.br (E.A.J.); 2 Discipline of Woman Health, Albert Einstein Israelite College of Health Sciences (FICSAE), Albert Einstein Israelite Hospital, São Paulo 05653-120, SP, Brazil; gycallado@gmail.com; 3 Department of Obstetrics, Paulista School of Medicine, Federal University of São Paulo (EPM-UNIFESP), São Paulo 04023-062, SP, Brazil; evelyntraina@gmail.com; 4 Department of Gynecology and Obstetrics, School of Medicine, Federal University of Rio de Janeiro (UFRJ), Rio de Janeiro 22240-003, RJ, Brazil; bragamed@yahoo.com.br; 5 Department of Maternal and Child Health, School of Medicine, Fluminense Federal University (UFF), Niterói 24070-090, RJ, Brazil; 6 Postgraduate Program in Applied Health Sciences, University of Vassouras, Vassouras 27700-000, RJ, Brazil; 7 Villa Sofia Cervello Hospital, University of Palermo, 90100 Palermo, Italy

**Keywords:** HTLV, vertical transmission, prenatal care, diagnosis, prevention, breastfeeding, pregnant women, neonatal infection

## Abstract

Human T-cell lymphotropic virus (HTLV), a retrovirus associated with severe conditions such as leukemia/lymphoma and myelopathy, exhibits variable global prevalence, with higher rates observed in regions such as northeastern Brazil and sub-Saharan Africa. While intrauterine transmission can occur via viral expression in placental tissue and contact with umbilical cord blood, the predominant route is vertical transmission through breastfeeding. Diagnostic testing, particularly serological screening with ELISA and confirmatory methods such as Western blot and PCR, is essential for early detection during pregnancy. The implementation of prenatal screening programs, as seen in Japan and Brazil, has proven effective in reducing vertical transmission by guiding interventions such as breastfeeding cessation in infected mothers. Beyond clinical implications, the psychosocial impact on affected pregnant women highlights the need for an interdisciplinary approach. Although the association between HTLV infection and adverse obstetric outcomes remains controversial, studies suggest increased risks of preterm birth, low birth weight, and other neonatal complications. Given the importance of early diagnosis and prevention, universal prenatal screening protocols represent a critical strategy to reduce viral transmission and its long-term consequences.

## 1. Introduction

Human T-cell lymphotropic virus (HTLV) is a retrovirus belonging to the Retroviridae family. It was the first oncogenic human retrovirus linked to infectious disease, discovered in the 1980s. HTLV exhibits tropism for T lymphocytes, in which it can induce cellular immortalization, thereby impairing their immunological function. HTLV is classified into four types, with HTLV-1 and HTLV-2 being the most prevalent. HTLV-1 primarily infects CD4+ lymphocytes, whereas HTLV-2 predominantly targets CD8+ lymphocytes. Both cause chronic infections with prolonged latency periods, ranging from 10 to 60 years. HTLV-1 is definitively associated with adult T-cell leukemia/lymphoma (ATL), with an estimated lifetime risk varying between 2 and 5%, depending on the population studied [1]. ATL is a refractory T-cell neoplasm that tends to be more aggressive in its acute form. Clinical manifestations may vary but often include generalized lymphadenopathy, hepatosplenomegaly, skin involvement, hypercalcemia, the presence of leukemic cells in peripheral blood, and opportunistic infections. HTLV-1 is also linked to HTLV-1-associated myelopathy (HAM), a chronic neurological disorder that affects fewer than 1% of infected individuals [2]. HAM results from progressive spinal cord demyelination and is clinically characterized by spastic paraparesis, urinary incontinence, and sensory disturbances that interfere with daily activities. Other conditions associated with HTLV-1 include infective dermatitis in children, uveitis, and, less frequently, arthralgia and polymyositis [3]. In contrast, no definitive association between HTLV-2 and human diseases has been established to date [2].

HTLV can be transmitted through three primary routes: mother-to-child (primarily via prolonged breastfeeding), sexual contact, and parenteral exposure to contaminated blood components or shared needles [3]. The principal route of vertical transmission (VT) of both HTLV-1 and HTLV-2 is breastfeeding. Vertical transmission can also rarely occur via transplacental/hematogenous routes or through very infrequent intimate contact during delivery; however, these are not considered common pathways of infection [4,5].

Studies indicate that serological screening and the cessation of breastfeeding in infected mothers can significantly reduce vertical transmission and associated complications [6]. However, in settings where breastfeeding is critical for infant nutrition and immunity, the risks and benefits of this intervention must be carefully evaluated. Although no clear differences in vertical transmission rates between HTLV-1 and HTLV-2 have been established, HTLV-1 is overwhelmingly more prevalent in the general population. Knowing the prevalence of the virus in the fertile age population, the rates of vertical transmission, and identifying the infection in pregnant women before childbirth are strategies that could assist in the development of protocols and policies for prenatal screening and effective interventions for disease prevention.

Screening for HTLV can be conducted using ELISA, Western blot (WB), line immunoassay (LIA), and polymerase chain reaction (PCR) tests. These all involve blood sampling but differ in methodology and purpose. ELISA detects HTLV antibodies but does not differentiate between virus types. WB, LIA, and PCR, although more expensive and time-consuming, can confirm infection and determine the HTLV type, thus guiding appropriate management. ELISA is typically used for initial screening due to its lower cost and faster turnaround time, while WB, LIA, and PCR are reserved for confirmation of positive results [7,8].

A study conducted by Imperial College London, in collaboration with Brazilian research groups including Fiocruz Bahia, estimated that public policies aimed at preventing HTLV-1 transmission via breastfeeding could prevent over 1000 pediatric infections annually in Brazil. Consequently, breastfeeding cessation is recommended for all HTLV-1-positive mothers, and in the absence of curative treatment or a vaccine, the most effective strategy against HTLV-1 remains the prevention of new infections [9].

The National Committee for Health Technology Incorporation (CONITEC) in Brazil recently recommended the incorporation of prenatal HTLV-1/2 testing into the public health system (SUS). The Products and Procedures Committee concluded that the test is safe and effective. Moreover, its implementation in the SUS would utilize already available resources, since tests for the detection of HTLV are already conducted outside the prenatal screening program [7].

Despite being described decades ago, HTLV infection remains largely unknown to the general public and healthcare providers. In clinical services caring for infected individuals, management should address not only disease risk but also patient education on transmission prevention [10,11]. That said, understanding the epidemiology and pathophysiology of HTLV, including its impact on pregnancy and breastfeeding, is essential for a better understanding and prevention of the disease. Therefore, we propose this work with the aim of conducting a narrative review of the literature on the subject.

This narrative review was based on a comprehensive literature search of studies published in the past two decades. Databases including PubMed/MEDLINE, SciELO, LILACS, and Embase were searched using the keywords ‘HTLV’, ‘vertical transmission’, ‘pregnancy’, ‘neonatal infection’, ‘diagnosis’, and ‘prenatal screening’.

## 2. Epidemiology

HTLV-1 is a human retrovirus with a heterogeneous global distribution and a higher epidemiological burden in endemic regions such as southwestern Japan, the Caribbean, sub-Saharan Africa, and South America, particularly Brazil, where it is estimated that between 800,000 and 2.5 million people are infected with the virus [12,13,14,15]. Due to limited population-based screening and the misattribution of symptoms to other conditions, many cases remain underdiagnosed. Globally, it is estimated that between 10 and 20 million people are affected by HTLV-1 [13,14]. In Brazil, prevalence rates vary by region, with the state of Bahia exhibiting the highest national rates. The city of Salvador alone accounts for nearly 40% of reported cases in the state [15].

Although most infected individuals remain asymptomatic, associated diseases typically emerge between the fourth and fifth decades of life. Approximately 2–10% of those infected develop severe clinical manifestations, including ATL and HTLV-1-associated myelopathy/tropical spastic paraparesis (HAM/TSP), often linked to a dysregulated immune response [13,14]. The absence of vaccines or curative treatment underscores the importance of continuous epidemiological surveillance, screening strategies, and public awareness to control infection and prevent HTLV-1-associated disease progression [13].

## 3. Modes of Transmission

HTLV-1 (human T-cell lymphotropic virus type 1) can be transmitted through multiple recognized routes, including horizontal (sexual and parenteral) and vertical (mother-to-child) transmission [14].

Sexual transmission occurs mainly through unprotected intercourse and is more efficient from men to women, especially in the presence of a high viral load in the seropositive partner. Parenteral transmission involves untested blood transfusions (transfusions performed prior to the implementation of mandatory HTLV screening), organ transplants, and the sharing of needles or syringes, being particularly relevant among intravenous drug users [16].

Vertical transmission is a significant public health concern, especially in endemic regions and among pregnant women. It occurs primarily through prolonged breastfeeding, which plays a significant role in the infection of infants [12,15,16]. This mode of transmission is mediated by infected T lymphocytes, particularly CD4+ T cells, present in breast milk. These cells are ingested during breastfeeding, traverse the infant’s intestinal mucosa, and infect immune cells [17]. Additionally, HTLV-1 infection of mammary epithelial cells may contribute to viral persistence in breast milk [18].

Other documented but less common vertical transmission routes include transplacental transmission, as evidenced by the detection of HTLV-1 in placental villous trophoblasts and umbilical cord blood, as well as perinatal transmission through contact with maternal blood in the birth canal. Although rare, postnatal non-lactational transmission—such as via saliva—has also been reported, though without consistent epidemiological evidence [6]. The risk of vertical transmission is influenced by several factors, including high maternal proviral load, the presence of HTLV-1-associated diseases such as ATL or HAM/TSP, co-infection with *Strongyloides stercoralis*, history of previously infected offspring, high HLA compatibility between mother and child, and unfavorable socioeconomic conditions [12].

Children infected early in life have an increased risk—up to 20%—of developing severe complications such as ATL, infective dermatitis, and HAM/TSP [16]. The rate of seroconversion in children varies according to feeding method: 11.2–23.7% with prolonged breastfeeding (>6 months), approximately 5.9–8.3% with short-term breastfeeding (<6 months), and 2.4–3.6% among those exclusively formula-fed [17]. Consequently, strategies such as prenatal screening, proviral load-based risk stratification, mode-of-delivery planning, and careful evaluation of breastfeeding risk–benefit profiles are essential to interrupt the viral transmission chain [16]. Preventing vertical transmission is critical not only to reduce the long-term neurological burden associated with HAM/TSP but also to avoid the development of ATL, a more frequent and potentially fatal malignancy that results from HTLV-1-mediated cellular transformation.

## 4. Clinical Manifestations

The clinical manifestations of HTLV-1 and HTLV-2 differ significantly. HTLV-1 is clearly associated with several diseases, most notably HAM/TSP and ATL. Additionally, HTLV-1 carriers have an increased risk of developing inflammatory and infectious diseases, including Sjögren’s syndrome, uveitis, arthropathies, leprosy, and infective dermatitis [19]. In contrast, HTLV-2 is less well studied and appears to be associated with a lower risk of clinical disease. However, recent studies suggest that individuals infected with HTLV-2 may be at increased risk of inflammatory and infectious conditions, although no specific diseases have been definitively attributed to HTLV-2 as they have been to HTLV-1 [19].

While HTLV-1 infection is often asymptomatic, relevant clinical manifestations can arise during pregnancy and the postpartum period. The principal neurological complication during this time is HAM/TSP—a chronic and progressive inflammatory disease [20]. Symptoms include progressive lower limb weakness with muscle stiffness, spastic gait, sphincter dysfunction (which may lead to urinary and fecal incontinence), and mild sensory changes such as decreased vibratory sensation, numbness, and tingling in the feet. In some cases, the upper limbs may also be affected, though the lower limbs are more commonly involved [20].

Although the global prevalence of HAM/TSP is low, it disproportionately affects communities in endemic regions such as Japan, the Caribbean, sub-Saharan Africa, and South America [20]. The risk of developing HAM/TSP among HTLV-1-infected individuals ranges from 0.25% to 4% and is up to three times higher in women [21]. During pregnancy, immunological changes may influence HAM/TSP progression, particularly in the immediate postpartum period. A Peruvian cohort study reviewed medical records of 1.439 HTLV-1-infected pregnant women and found that 15 of them reported the onset or worsening of HAM/TSP symptoms—such as walking difficulties and severe leg weakness—after delivery. Among these, five women had already reported symptoms during prenatal care, which worsened postpartum, significantly impairing basic activities such as newborn care. The remaining ten only developed symptoms after delivery. Most of these women reported leaving work and experiencing progressive mobility loss, with one ultimately requiring a wheelchair [20].

The early symptoms of HTLV-1 infection—such as fatigue and lower limb weakness—are often misattributed to pregnancy or postpartum fatigue. Furthermore, socioeconomic barriers and a general lack of awareness about HTLV-1 among healthcare providers and the population in endemic areas hinder early diagnosis [10].

Some evidence suggests that pro-inflammatory changes caused by the virus may hypothetically lead to adverse obstetric outcomes, although the data remain inconclusive. A Peruvian cohort study evaluated 58 children infected with HTLV-1 and found that, when compared to a control group (42 uninfected children born to seropositive mothers), they had higher rates of low birth weight (41% vs. 19%), prematurity (20% vs. 12%), and neonatal hospitalizations (18% vs. 12%) [22]. An Iranian study evaluated the impact of HTLV-1 on the fertility of infected women and found no differences in embryo quality or implantation success in assisted reproduction cycles between the infected and control groups. The rates of miscarriage and preterm delivery were also similar, suggesting that HTLV-1 may not significantly impair fertility [23].

Regarding fetal outcomes, most infants infected with HTLV-1 remain asymptomatic for many years but are at increased risk of developing associated diseases such as ATL and HAM/TSP, especially when infected early in life [24]. Although early psychomotor development is usually unaffected, infected children have higher rates of clinical signs such as leg weakness, urinary dysfunction, eczema, dermatitis, and lower limb hyperreflexia [24]. Early infection is also associated with HTLV-1-associated infective dermatitis (IDH) [25]. Table 1 shows the maternal and perinatal outcomes associated with HTLV-1 infection.

## 5. Diagnosis

Early diagnosis of HTLV infection during pregnancy is essential for the prevention of vertical transmission [26]. Accurate identification enables appropriate clinical management and the implementation of preventive measures during pregnancy and the postpartum period, thereby reducing the risk of transmission to the newborn [16]. Comprehensive prenatal care facilitates the detection of infected pregnant women and the planning of interventions to reduce risks for both mother and child [27].

Several studies support universal prenatal screening. In Japan, universal mandatory screening of pregnant women using the ELISA test has been national policy since 2010, resulting in a significant reduction in vertical transmission following its implementation. This pioneering experience serves as a model for other high-prevalence countries. In Europe, a study on the economic impact conducted in the United Kingdom demonstrated that HTLV-1 prenatal screening can be profitable, particularly in areas with a higher incidence of the infection [13]. Even without economic impact studies, regional surveys in Brazil show a significant number of infected pregnant women, particularly in endemic regions such as Bahia. This highlights the importance of implementing screening policies in health facilities offering prenatal care. These findings are supported by epidemiological information presented in reviews and meta-analyses covering Brazil and other Latin American and Caribbean countries [28].

Initial HTLV-1/2 screening during pregnancy is typically performed using serological tests due to their practicality, affordability, and high sensitivity. The enzyme-linked immunosorbent assay (ELISA) is widely considered the standard first-line screening tool in public policy such as those in Japan. This method has demonstrated high sensitivity in pregnant women, without significant interference from gestational physiology [29,30]. Several studies confirm ELISA’s reliability in detecting HTLV-infected pregnant women, making it an ideal foundation for screening in high-prevalence settings.

Chemiluminescent immunoassay (CLIA) is an increasingly used automated technique that offers enhanced accuracy and reduced processing time. A recent meta-analysis demonstrated CLIA’s high precision for screening purposes. Studies also indicate that CLIA results remain stable during pregnancy [31], supporting its use in prenatal programs. Moreover, the automated nature of CLIA makes it scalable, and it has been implemented successfully in public health systems, including in England and several Brazilian states. In countries such as Japan, CLIA is used as the initial screening method, followed by LIA for confirmatory testing. If LIA results are indeterminate, PCR is subsequently employed to establish the diagnosis, in accordance with national guidelines.

Although less frequently employed, particle agglutination is used in Brazil in diagnostic algorithms to resolve indeterminate ELISA results, especially in areas with limited access to advanced testing infrastructure [32]. ELISA kits currently used in Brazil and Japan for HTLV-1/2 screening typically detect total antibodies (IgG and, in some cases, IgM) against viral envelope (gp46) and core (p24) proteins. However, unlike CMV or EBV diagnostics, these assays do not distinguish between latent infection and viral reactivation, as HTLV infection remains persistently detectable once seroconversion occurs. While simple to perform, its lower sensitivity restricts its role to a complementary test. Indirect immunofluorescence (IF) is also used as a confirmatory method, particularly when WB results are indeterminate [32]. Despite good specificity, IF use is limited by its need for specialized infrastructure and equipment. WB remains the gold standard for confirmatory testing following a positive screening result, due to its high specificity in detecting antibodies against viral proteins [33]. However, WB is more expensive and slower, limiting its scalability in large screening programs.

Qualitative PCR (nPCR) is used to detect HTLV DNA directly, particularly in cases with inconclusive serological results or when distinguishing between HTLV-1 and HTLV-2. Quantitative PCR (qPCR), in turn, measures the proviral load (PVL), which is associated with both vertical transmission risk and the development of HTLV-related diseases. Recent studies have validated the use of a duplex PCR method, which reliably differentiates HTLV-1 from HTLV-2 with excellent sensitivity and specificity. Although latex particle-based rapid tests have lower sensitivity, they may serve as initial screening tools in remote areas lacking laboratory infrastructure [34].

Recent research has also investigated the effectiveness of multi-epitope recombinant proteins for serological diagnosis, with promising preliminary results in terms of sensitivity and specificity. These developments may represent a future advance in HTLV screening [35]. To date, there is no consistent evidence in the literature establishing a correlation between HTLV-1 infection and specific prenatal ultrasound findings. Obstetric ultrasound remains crucial for routine pregnancy monitoring, but no morphological parameters have been identified to indicate HTLV-1 infection or predict vertical transmission [12,36]. Cohort studies conducted in endemic Brazilian regions have not reported any specific ultrasonographic features related to HTLV-1 infection, reinforcing the understanding that this infection does not manifest with morphological abnormalities detectable via prenatal imaging.

Economic impact studies suggest that automated prenatal testing methods, such as CLIA, may be cost-effective and feasible in both high- and middle-income countries. However, logistical and infrastructural challenges remain a barrier to national standardization. In many areas, confirmatory tests are unavailable, and the lack of reliable rapid tests hinders early diagnosis in remote regions [37]. Confirming suspected cases is essential to avoid false positives, which requires access to WB, PCR, and other specialized diagnostic tools that are still limited in Brazil’s public health system.

To optimize diagnosis and clinical management during pregnancy, clinical algorithms integrating serological, molecular, and clinical data have been proposed. These tools help inform key decisions, including the cessation of breastfeeding, neonatal monitoring, and genetic counseling [38]. Table 2 shows the laboratory tests used for the diagnosis of HTLV.

## 6. Prenatal Care in HTLV Infection

Human T-cell lymphotropic virus (HTLV) infection represents a growing public health concern, particularly in the context of pregnancy and vertical transmission. Globally, Brazil has the highest absolute number of individuals infected with HTLV. Accordingly, efforts have been made to develop strategies for monitoring and supporting HTLV-positive pregnant women, aiming to promote early diagnosis and prevent vertical transmission [39,40].

### 6.1. Screening and Diagnosis

Early diagnosis of HTLV infection during prenatal care is a safe and effective strategy for preventing vertical transmission and minimizing its consequences [40]. In Brazil, the Ministry of Health—following recommendations from CONITEC—has incorporated HTLV-1/2 screening into the SUS. Testing is ideally conducted during the first prenatal visit, with ELISA used for initial screening and Western blot or PCR reserved for confirmatory purposes. These tests were already available in other settings, which facilitated their integration into prenatal care without significant additional costs [40].

Japan serves as a reference model for comprehensive prenatal HTLV care. Since 2010, its nationwide screening program recommends that pregnant women be tested before 30 weeks of gestation. In cases of positive screening, confirmatory testing is performed, followed by PCR in indeterminate cases. Once infection is confirmed, patients receive counseling and are monitored to ensure they are informed about managing both maternal infection and neonatal care [29]. Maternal diagnosis during pregnancy is essential for implementing targeted interventions to reduce the risk of neonatal infection, particularly in terms of breastfeeding guidance and other neonatal management strategies [16,41].

### 6.2. Counseling and Interdisciplinary Care

HTLV diagnosis during pregnancy has psychosocial implications beyond clinical management. Studies have shown that not breastfeeding can trigger feelings of guilt, fear, stigma, and sadness [39,41]. Women who choose exclusive formula feeding—or even short-term breastfeeding—are more vulnerable to anxiety and depression, highlighting the need for emotional support and professional counseling [41].

Thus, clinical management of HTLV during pregnancy must also address mental health. Multidisciplinary care—integrating physicians, nurses, psychologists, and social workers—is essential. This approach, as implemented at CHTLV (HTLV Reference Center in Bahia), ensures continuous and compassionate care for seropositive pregnant and postpartum women, as well as their support networks [39].

### 6.3. Breastfeeding and Vertical Transmission

Breastfeeding is the primary route of HTLV vertical transmission, and infection risk in infants is directly related to maternal viral load and breastfeeding duration. Brazilian studies have shown that 53% of infants breastfed by infected mothers tested positive for HTLV by two years of age—approximately 18 times higher than among non-breastfed infants [39]. Duration of breastfeeding is the key determinant of transmission risk. Infection rates are <2.5% for breastfeeding durations under 3 months, approximately 3.9% for 3–6 months, and 20.3% for durations exceeding 6 months [16].

Although no evidence suggests that exclusive breastfeeding for less than 90 days increases the risk of HTLV-1 transmission compared to exclusive formula feeding, short-term breastfeeding may lead to challenges with subsequent weaning [16]. Thus, the main recommendation is to avoid breastfeeding. In cases where exclusive formula feeding is not feasible, breastfeeding should not exceed 90 days and should be discontinued as early as possible [16,29].

### 6.4. Delivery and Intrapartum Management

Although delivery is not the main route of HTLV transmission, it constitutes a potential vertical transmission pathway due to neonatal exposure to maternal blood. Accordingly, certain precautions are recommended to reduce exposure and associated risks [16]. In most cases, the delivery method follows standard obstetric criteria, and vaginal delivery is not contraindicated based solely on HTLV status [16,39]. Regarding umbilical cord management, immediate clamping is recommended for all HTLV-positive mothers to reduce the risk of transferring infected lymphocytes to the neonate [39].

### 6.5. Additional Strategies

**Figure 1:**
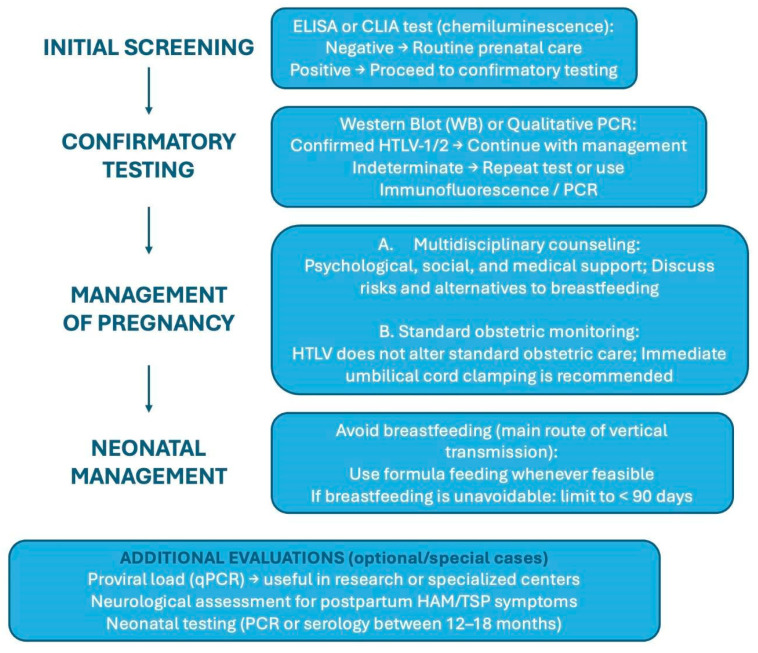
Screening, confirmatory testing, and pregnancy/neonatal management of HTLV.

The use of antiretrovirals for HTLV is still under investigation. In vitro studies have shown that zidovudine (AZT) and interferon-alpha exhibit activity against the virus and appear safe for use during pregnancy. Moreover, experimental models and small clinical reports suggest that post-exposure prophylaxis (PEP) in neonates may reduce transmission in cases of significant intrauterine or peripartum exposure [16]. However, these therapeutic approaches are not yet supported by robust clinical evidence. Figure 1 summarizes the screening, confirmatory testing, and pregnancy/neonatal management of HTLV.

## 7. Conclusions

According to the most recent literature, human T-cell lymphotropic virus (HTLV) infection occurs primarily through vertical transmission via breast milk. Nevertheless, intrauterine transmission remains a significant concern, as evidence shows HTLV-1 expression in placental villous tissue in approximately 50% of infected pregnant women studied and to a lesser extent in umbilical cord blood [6].

The prevalence of HTLV infection among pregnant women varies by region. In Brazil, HTLV-1 is more prevalent in the northeast, while HTLV-2 is more commonly found in the south. Overall, HTLV-1 has higher global prevalence than HTLV-2 [30,42]. Sub-Saharan Africa is also considered an endemic region, especially for HTLV-1, showing patterns similar to those observed in Brazil [43].

Screening programs have proven fundamental for early identification and intervention to prevent vertical transmission. A successful example is the program implemented in Japan, which, upon detecting infection in pregnant women, recommends interventions such as breastfeeding substitution—a measure shown to substantially reduce mother-to-child transmission [13,30]. Serological screening is a reliable approach, as pregnancy does not affect the accuracy of diagnostic testing for either HTLV type [31].

During pregnancy, HTLV-1 infection generally remains stable, particularly regarding proviral DNA levels. Therefore, the virus is not believed to be directly associated with obstetric or perinatal complications. However, a rise in viral load has been observed in the postpartum period, possibly due to immunological shifts typical of the puerperium. This increase may help explain the heightened risk of transmission through breastfeeding [42,44].

HTLV-1 infection during pregnancy is, in most cases, asymptomatic for both mother and newborn. Nevertheless, the risk of vertical transmission through breastfeeding remains high [45]. Although rare, both infected mothers and children may later develop HAM/TSP, a severe neurological complication driven by the host immune response to the virus, which can significantly impair quality of life [46]. While neuropsychomotor development is generally preserved in infected children, higher incidences of clinical symptoms—such as muscular weakness and dermatitis—have been reported [22].

Long-term data on the impact of HTLV-1 infection, particularly among pregnant women and neonates, remain limited. In light of this gap, the present review underscores the importance of implementing prenatal screening programs to prevent the main mode of vertical transmission: breastfeeding. Given the still-incomplete understanding of the virus’s effects on mothers and infants, early diagnosis is critical for guiding effective preventive strategies. Furthermore, the stigma associated with HTLV, compounded by the lack of information, underscores the need for multidisciplinary care and the development of new health education strategies.

## Figures and Tables

**Table 1:** Maternal and fetal/child clinical outcomes associated with HTLV-1 infection.

Category	Clinical Manifestations
Maternal	HTLV-1-associated myelopathy/tropical spastic paraparesis (HAM/TSP), progressive lower limb weakness, muscle stiffness, spastic gait, sphincter dysfunction.
Fetal/Child	Increased risk of developing HAM/TSP, lower limb weakness and hyperreflexia, HTLV-1-associated infective dermatitis (IDH), low birth weight (suggestive), prematurity (suggestive).

**Table 2:** Laboratory tests used for the diagnosis of HTLV.

Test	Method	Purpose
ELISA	Serology	Initial screening
CLIA (chemiluminescence)	Serology	Initial screening
Particle agglutination	Serology	Initial screening
Western blot (WB)	Serology	Confirmatory
Line immunoassay (LIA)	Serology	Confirmatory
Indirect immunofluorescence	Serology	Confirmatory (alternative)
Qualitative PCR (nPCR)	Molecular	Detection of viral DNA
Quantitative PCR (qPCR)	Molecular	Proviral load quantification (PVL)
Duplex PCR	Molecular	Differentiation between HTLV-1 and HTLV-2
Recombinant multi-epitope	Serology	Diagnostic method under development

## Data Availability

The data presented in this study are available on request from the corresponding author.
